# More mental health problems after divorce in couples with high pre-divorce alcohol consumption than in other divorced couples: results from the HUNT-study

**DOI:** 10.1186/1471-2458-13-852

**Published:** 2013-09-17

**Authors:** Kamilla Rognmo, Fartein A Torvik, Mariann Idstad, Kristian Tambs

**Affiliations:** 1 Norwegian Institute of Public Health, Division of Mental Health, PO BOX 4404 NydalenN-0403, Oslo, Norway

**Keywords:** Divorce, Alcohol use, Mental health, Selection effects, Social causation

## Abstract

**Background:**

Divorce is associated with mental health problems, and heavy drinking is related to higher risk of divorce. Less is known about the effects of divorce in couples where one or both drinks heavily. There are, however, reasons to expect different consequences of divorce in heavy risk using couples compared to other couples. Spouses of abusers may experience the divorce as a relief, whereas abusers may find it extra difficult to be left single. The aim of the study is to compare the effect of divorce on mental health in heavy drinking couples to the effect in couples who drink less.

**Methods:**

Registry data were matched with data from the Nord-Trøndelag Health Study (HUNT 1 (T1) and 2 (T2)), enabling longitudinal analyses of approximately 11,000 couples. Interaction terms between 1) alcohol use on T1 and divorce between T1 and T2 (11 year time lag), and 2) alcohol use on T1 and time since divorce at T2 for all divorced couples were tested to examine changes in mental health between T1 and T2 for a) *spouses* of high-risk compared to low-risk users, and b) high-risk compared to low-risk *users themselves*. Data were analyzed using multivariate analysis of variance.

**Results:**

There was a general effect of divorce on change in mental health between T1 and T2. We observed a significantly stronger worsening in mental health in female high-risk users and their spouses than in divorced low-risk users and their spouses. The results also suggest that the strain after divorce lasts longer in women with a high alcohol consumption and their spouses.

**Conclusions:**

Divorce seems to affect couples where one or both drink heavily more than couples with a low consumption. Also there was some evidence of a slower healing of mental health problems after divorce in alcohol exposed couples than in other couples. The results only reached significance for female high consumers and their spouses, but due to limited statistical power, safe conclusions about gender specific effects cannot be drawn.

## Background

The relationship between heavy drinking and divorce has long been recognized [1,2]. Alcohol abusers have approximately 20% higher risk of getting divorced compared to the risk in the normal population [3]. Nevertheless, little is known about the effect of divorce on the mental health of individuals from marriages with alcohol abuse. In contrast, the effects of divorce in the general population have been extensively studied [4], and results reveal that although the risk of mental health problems is higher some years prior to the divorce, the levels seem to peak in the period following the incident [5]. These findings have been explained by a combination of the health selection model [6-8] – in which mentally troubled individuals are less likely to stay married – and the social causation model – stating that the mental health problems are caused by the adversities related to the divorce, such as emotional stress, unhealthy lifestyle, less social support and fewer material resources [9]. However, findings regarding the effects of divorce in the general population may not be fully valid for couples where one or both drinks heavily. To our knowledge, the effects of divorce on mental health specifically among heavy drinkers or among spouses of heavy drinkers have not been studied previously.

It is reasonable to assume that the change in mental health after a divorce may vary for the alcohol abuser and non-abusing spouse. Numerous studies have provided evidence stating that being married to an alcohol abuser is related to adverse outcomes – such as higher risk of experiencing violence [10,11], poor physical health [12] and possibly poor mental health, although the relationship between alcohol abuse and spousal mental health is less clear [12-19]. The adverse factors associated with being married to an alcohol abuser may give reason to believe that divorcing the abuser may cause a decrease in mental health problems for the non-abusing spouse, rather than an increase, as usually observed in the general population after a divorce. One study found that women divorcing problem drinking men exhibited less frequent drinking, less heavy drinking and less alcohol related problems after the divorce [20]. However, the adversities related to divorce in the general population [9] may also follow for spouses of alcohol abusers. The key question is whether getting rid of the problems related to the marriage with an alcohol abuser trumps the adversities related to divorce in general.

According to the health selection hypothesis, heavy alcohol consumers are less desirable partners, reflected in the lower likelihood of getting married and the higher likelihood of divorce [21]. There seems, however, to be a protective effect of marriage – as marriage reduces alcohol consumption [22,23], enhances remission rates and deters problem drinking [24]. These factors may give the alcohol abusers greater incentive to stay married and higher risk of mental health problems if divorced. Heavy alcohol consumers and their spouses have increased risk of marital dissatisfaction [25,26], and both heavy alcohol use and marital dissatisfaction are related to marital dissolution [27]. However, individuals reporting high levels of marital dissatisfaction before the divorce have been shown to have less mental health problems [28] and higher life satisfaction [29] after the divorce, which may imply that divorce in couples suffering from alcohol abuse, as alcohol abuse is related to marital dissatisfaction, may be related to a decrease in mental health problems.

The change in mental health post-divorce may depend upon several factors, of which one is gender. Although anxiety and depression are more prevalent in women [30], several studies suggest graver post-divorce consequences for men than for women [31,32]. The effect of change in mental health problems also seems to depend upon time since the divorce. Mental distress has been found to peak around the time of the divorce [5], and the symptom levels are elevated for many years after the divorce.

Knowledge about the effects of divorce on mental health in couples where one spouse or both drinks heavily is needed, but to our knowledge, no previous study has investigated the effects of divorce on change in mental health in this particular group. In the present study, we aim at comparing the change in mental health after a divorce between a) spouses of heavy drinkers and spouses of normal consumers, and between b) heavy drinkers and normal consumers. A tentative hypothesis would be that mental health worsens more after a divorce among high risk users than among other divorced people. Persons divorcing high risk users may or may not have worsened mental health after the divorce, but we expect the worsening to be less severe than in other divorced couples. All analyses are stratified by gender. In order to avoid confounding from the other spouse’s heavy drinking, we adjust for drinking in the spouse (when investigating the subject’s own change in mental health) or the subject’s drinking (when investigating the spouse’s change in mental health), alongside demographic factors. In addition, we investigate to what extent change in mental health from before to after a divorce depends upon time since divorce, that is, how fast the detrimental effects of divorce is healing. Finally we test whether such a healing process is faster or slower among people breaking up from a marriage burdened by heavy drinking than in other divorced people.

## Methods

### Sample

Registry data, combined with longitudinal data from the Nord-Trøndelag Health Study (HUNT 1 and 2) is used in the present study. HUNT 1 was carried out in 1984–86, and HUNT 2 in 1995–97, moving from municipality to municipality, and with the same order of municipalities in HUNT 1 and 2. The entire population of Nord-Trøndelag County, Norway, aged 20 or more, was invited to participate both times, with a eleven year time lag between T1 and T2. Both waves included a health examination and two questionnaires. The first questionnaire (Q1) was enclosed with the invitation letter and returned at the examination site, whereas the second (Q2) was distributed at the examination site and returned by prepaid mail. Data from HUNT 1 and 2 were matched with data from public registries administered by Statistics Norway, using the 11-digit personal identification number assigned to every Norwegian citizen as the matching key. This enabled the identification of married and divorced or separated couples, and the time of divorce/separation. In total, 86,404 persons were invited to participate in HUNT 1. Of these, 77,212 (89.4%) responded to Q1 and 63,943 (74.0%) responded to both Q1 and Q2. 93,898 were invited to participate in HUNT 2. Of these 65,237 (69.5%) returned Q1 and 55,313 (58.9%) returned both Q1 and Q2. All analyses in this study are based on data from persons who completed both Q1 and Q2. For one analysis, in which we checked for the baseline differences in mental health between people who later divorced and those who stayed married, there were 27,047 heterosexual couples in which at least one of the spouses participated in HUNT 1. In the analyses of women’s change in mental health (either as a result of spousal alcohol consumption or own alcohol consumption) the woman needed to have participated in both HUNT 1 and 2 and the man only in HUNT 1. Correspondingly, when investigating men’s change in mental health, the man needed to have participated in both HUNT 1 and 2, and the women needed only to have participated in HUNT 1. This rendered 14,985 couples (62.7% of all couples who were invited to HUNT 1, where at least the woman was invited to HUNT 2) with the first combination of participants (12,850 with complete data) and 13,010 couples (60.3% of all couples who were invited to HUNT 1, where at least the man was invited to HUNT 2) with the latter combination of participants (10,915 with complete data). The data file was arranged with couples as the analyzing unit. Husbands’ variables were located in the first part of the record, and wives’ variables in the last part of the record. Unfortunately, there were not enough same sex or cohabiting, unmarried couples in the data to perform meaningful comparative analyses, and consequently, these were excluded from the sample.

### Ethics

The HUNT-Study has been approved by the Regional Committee for Medical and Health Research Ethics for Central Norway and has been performed in accordance with the ethical standards laid down in the 1964 Helsinki declaration. All respondents gave their written consent for their data to be used for research purposes.

### Measures

#### Registry data

##### Divorce/separation

Data on year of divorce/separation were provided by governmental public registries. Respondents getting divorced/separated during the ten years prior to their participation in HUNT 2 were categorized as divorced. That is, respondents participating in HUNT in 1984, and then again in 1995, were registered as divorced if the divorce occurred within the time period 1985 to 1994. Divorce was observed in the period 1986–1995 for subjects participating in HUNT in 1985 and 1996, and in the period 1987–1996 for subjects participating in 1986 and in 1997. Because most separated spouses will eventually divorce, all divorced/separated persons will be referred to as divorced from hereon. For the purpose of the “time since divorce analyses”, all divorced individuals were categorized according to number of years since divorce from T2: 1–3 years, 4–7 years and 8–10 years since divorce. The men’s participation date and registered divorce information were used where available. In case of missing data, the woman’s information was used.

##### Demography

Registry data on the highest completed education were provided by public registries. Valid registry data from 1985 were available for 98.7% of the participants. Registry data on education in 1980 or 1990 were used for the remainder of the participants. There were nine educational categories in the registry data: 1) no education, 2) lowest public school, seven years (older cohort), 3) obligatory public school, nine years (younger cohort), 4) obligatory public school, plus one to two years of vocational school, 5) high school, 12 years, 6) public school, plus four years of vocational school, 7) one to four years at university or college, 8) five to seven years at university or college, 9) doctoral degree. Education was recoded into four groups: elementary school (categories 1–3, men: 38.9%, women: 42.5%), some secondary education (category 4, men: 26.5%, women: 38.9%), completed secondary education (categories 5–6, men: 22.2%, women: 8.1%), and college/university (categories 7–9, men: 12.4%, women: 10.5%). Registry data on age were also provided by public registries, and used as a linear covariate. Mean age was 48.3 for men, and 46.1 for women.

#### Questionnaire data

##### High-risk alcohol use

Alcohol use was measured by three items: “How often did you drink alcohol over the last 14 days?” (Total abstainer, 0 times, 1–4 times, 5–10 times, 10 times or more), “If you drank alcohol during the last 14 days, did it make you feel influenced by alcohol on any occasion?” (No, Yes), and “Have there been periods in your life during which you have drunk excessively or at least a bit too much?” (No, Not sure, Yes). Having reported to have been influenced by alcohol, and yet responded to be total abstainers or not having consumed alcohol within the last 14 days was coded “No” on the influenced item. The three items were standardized, before summed into an index, of which the top 13.1% of male and top 9.3% of female respondents were categorized as high-risk users. The distribution of the scores necessitated the chosen cut-off values, as there was a clustering of scores just below the cut-off points. The somewhat liberal definition of female high-risk users was warranted due to small groups in the “time since divorce-analyses”. All respondents scoring below the cut-off were categorized as low-risk users. 87.5% of the high-risk women and 30% of the low-risk women reported to have been drinking 1–4 or more times during the past 14 days. 67.8% of the high-risk women and 0.2% of the low-risk women reported to have been influenced by alcohol during the last 14 days, and 51.4% of the female high-risk users and 4.5% of the low-risk users reported to have drunk excessively at one point during their lives. 99.4% of the male high-risk users and 52.8% of the male low-risk users reported to have been drinking 1–4 or more times during the past 14 days. 87.3% of the high-risk men and 16.2% of the low-risk men reported to have been influenced by alcohol during the last 14 days, and 91.4% of the male high-risk users and 17.7% of the low-risk users reported to have drunk excessively at one point during their lives.

##### Mental health

Nine items measuring mental health were included in both HUNT 1 and HUNT 2, asking about nervousness, sleeping problems, feeling calm and good, the use of tranquilizers, feelings of happiness, feeling lonely, feeling strong and fit or tired or worn out, life satisfaction, and impairment due to mental health problems. Weights estimated in previous studies based on the same material [33,34] were assigned each item in order to maximize the correlation between a summative index based on the nine items (from now on referred to as MH) and the Hopkins Symptom Check list-25 [35], giving the intercorrelation r = 0.83 [34]. The items and their assigned weights are described in detail elsewhere [34]. Separate composite scores for mental health at T1 (MH1) and T2 (MH2) were computed. Cronbach’s alpha for MH was 0.81 for males and 0.85 for females at T1, and 0.81 for males and 0.82 for females at T2.

One of the nine MH items was not included in HUNT 2 for persons older than 69 years, who completed a special questionnaire version for the elderly. For this group, which included 5755 persons (22.2%), missing values for the missing item were imputed (see below)*.*

### Missing values

After treating data as described above, the registry data did not have any missing values. SPSS Missing Value Analysis (MVA), Expectation Maximization (EM) [36] was used to impute values for the questionnaire data. Here values from the valid items are used to predict values for items with missing data. Imputations were made for respondents with valid data for minimum 50% of the items of each set of items. Prior to imputation, missing data on the “influenced by alcohol” item were recoded to zero if the respondent reported to be abstainers or not having consumed alcohol within the last 14 days. Data gained by imputing missing values were calculated based on the respondents returning a total of four questionnaires, two in HUNT 1 and two in HUNT 2. Imputing the alcohol items reduced missing values from 9.4% to 1.1% for the male respondents and from 21.3% to 2.4% for the female respondents. Missing values for MH1 were reduced from 6.2% to 0.9% for the men and from 9.6% to 1.1% for the women. Missing values for MH2 were reduced from 14.4% - including those responding to the questionnaire version missing one item, and of which 11.8% had one missing item - to 1.1% for men, and from 18.5% - of which 14.9% had one missing item - to 1.3% for women.

### Statistical analyses

In order to describe group differences in mental health at baseline (T1), multivariate analyses of variance (General Linear Model, SPSS) were conducted, with MH1 as the outcome variable. Two categorical predictor variables were generated, one combining divorce/staying married between T1 and T2 and spousal alcohol use (low-risk/high-risk) at T1, the other combining divorce/staying married with own alcohol use. The reference groups included persons still married to low risk drinkers or persons still married who were themselves low risk drinkers. In the analysis comparing the categories combining divorce and (in one analysis spousal, in another own) alcohol we adjusted for the other spouse’s alcohol use together with age and education of the person whose mental health was examined. We also stratified by gender.

In order to investigate change in mental health between T1 and T2, multivariate analyses of variance (General Linear Model, SPSS), stratified by gender, were conducted with mental health at T2 as the outcome variable and divorce and partners’ and subjects’ own high-risk alcohol use as predictor variables together with baseline (T1) mental health. The spouses’ and/or subjects’ age and education were also entered as covariates. When adjusting for mental health at T1 the results express change in mental health between the two time points. The analyses were conducted in three steps. First, the main effects were estimated. Second, the interaction terms between divorce and partners’ or subjects’ high-risk use were entered into the model. Interaction terms were entered one at the time. Third, significant interaction effects were further investigated in stratified samples.

In a second set of analyses, including only divorced persons, the effect of number of years since divorce was entered as a predictor variable instead of divorce per se. The divorced individuals were categorized according to number of years since divorce, 1–2 years, 3–4 years or 5–7 years since divorce. The statistical models were otherwise identical to the models described above, comparing high-risk drinkers and spouses of high-risk drinkers with persons from couples without high-risk drinkers.

## Results

### Mental health at T1

Mean difference in mental health at T1, adjusted for age and education, between groups categorized by later divorce/still married at T2 and spousal or own high-risk alcohol use, are displayed in Tables 1 and 2. All groups differed significantly from the reference groups – married persons with low risk spousal or own alcohol use. The results show that those who will get divorced - regardless of spousal or own drinking – have higher mental health problem scores than those who will stay married until T2 (Tables 1 and 2). The baseline mental health scores are particularly poor for the to-be-divorced high-risk users (Table 2).

**Table 1:** Baseline mental health for spouses

	**Women**		**Men**	
	**Mean**^**a**^	**p**	**Mean**^**a**^	**P**
***Divorce and spousal high-risk use***				
To be divorced – partner high-risk use	.20	<.001	.17	.002
To be divorced- partner low-risk use	.23	<.001	.28	<.001
Remain married – partner high-risk use	-.05	.037	-.02	.037
Remain married– partner low-risk use	-.10	Ref	-.09	Ref

^a^ Adjusted group means in standard deviations.

Sample includes only couples with complete data.

Mental health scores at T1 are standardized.

Group differences, analyzed by ANOVA, in mental health at T1 according to spousal alcohol use at T1 and later divorce, adjusted for age and education. The estimates are adjusted group means on a standardized outcome variable, with a sample mean of 0.

**Table 2:** Baseline mental health for subjects

	**Women**		**Men**	
	**Mean**^**a**^	**p**	**Mean**^**a**^	**P**
***Divorce and subjects’ high-risk use***				
To be divorced – high-risk use	.43	<.001	.53	<.001
To be divorced – low-risk use	.20	<.001	.21	<.001
Remain married – high-risk use	.10	<.001	.19	<.001
Remain married – low-risk use	-.12	Ref	-.12	Ref

^a^ Adjusted group means in standard deviations.

Sample includes only couples with complete data.

Mental health scores at T1 are standardized.

Group differences, analyzed by ANOVA, in mental health according to subjects’ own alcohol use at T1 and later divorce, adjusted for age and education. The estimates are adjusted group means on a standardized outcome variable, with a sample mean of 0.

#### Main effects of divorce and high-risk use on change in mental health problems between T1 and T2

Multivariate analyses of variance, stratified by gender, were conducted with mental health at T2 as the outcome variable and divorce between T1 and T2 and spouses’ or own high-risk alcohol use as predictor. Effects were adjusted for age, education and mental health at T1. The results are displayed in Table 3. Compared to persons still married, persons who divorced between T1 and T2 had a significantly worsened mental health (0.27 of a standard deviation (SD) for women, 0.35 SD for men. Own alcohol high risk use was associated with significantly worsened mental health (0.09 SD for women and 0.10 SD for men), spousal high-risk use was not.

**Table 3:** Change in mental health between T1 and T2

	**Women**				**Men**			
	**N**	**B**^**b**^	**CI**	**p.**	**N**	**B**^**b**^	**CI**	**p.**
*Divorce*				<.001				<.001
Yes	634	.27	.21–.34	<.001	560	.35	.28–.43	<.001
No	12119	0			10198	0		
*Partner’s high-risk use*				.138				.742
Yes	1649	.03	-.01–.08	.138	1067	.01	-.05–.07	.742
No	11104	0			9691	0		
*Own high-risk use*				<.001				<.001
Yes	1169	.09	.04–.14	<.001	1386	.10	.05–.15	<.001
No	11584	0			9372	0		
*Education*				. < 001				.123
Elementary school	4720	.11	.06–.16	<.001	3465	.07	.01–.12	.016
Started high school	5442	.05	-.00–.09	.068	3046	.05	-.01–.10	.098
Finished high school	1125	.03	-.04–.09	.403	2730	.04	-.01–.07	.127
University	1466	0			1517	0		
*Age*		.01	.01–.01	<.001		.00	.00–.01	<.001
*Mental health T1*		.54	.52–.55	<.001		.49	.47–.51	. < 001

^a^ Mental health at T2 is standardized.

^b^ B = adjusted group difference in fractions of standard deviations.

Analysis of variance of divorce and spouses’ and subjects’ own alcohol use at T1 on change in mental health from T1 to T2, adjusted for age, education and mental health at T1^a^.

#### Interaction effects between divorce and alcohol use

Interaction terms between divorce and a) spouses’ high-risk alcohol use, and b) subjects’ own high-risk alcohol use were specified. The estimates were adjusted for age, education and mental health at T1. The results are displayed as simple effects analyses stratified by gender in Table 4 and 5. Two interaction effects came out significant. As shown in Table 4, men divorcing female high-risk users experienced a greater increase in mental health problems (0.54 SD among divorced vs. -.01 SD among not divorced) compared to men divorcing low-risk users (0.27 SD vs. -0.03 SD). Also, female high-risk users experienced a greater increase in mental health problems after getting divorced than did low-risk users (Table 5). The same but non-significant (p = 0.108) trend was observed for male high-risk consumers.

**Table 4:** Change in mental health for spouses of high and low-risk alcohol users

		**High-risk spouse**			**Low-risk spouse**			
		**N**	**Mean**^**a**^	**CI**	**N**	**Mean**^**a**^	**CI**	**p**
*Women*								.394
	Divorced	135	.21	.06-.35	499	.26	.18-.33	
	Not divorced	1514	.03	-.01-.07	10605	-.04	-.05- -.02	
	Total	1649	.04		11104	-.02		
*Men*								.009
	Divorced	121	.54	.38-.70	439	.27	.19-.36	
	Not divorced	946	-.01	-.05-.07	9252	-.03	-.05- -.01	
	Total	1067	.07		9691	-.01		

^a^ Mental health at T2 is standardized.

Interaction effects, shown in adjusted group means, between divorce and spouses’ alcohol use on change in subjects’ mental health. Effects are adjusted for age, education, subjects’ own alcohol use, and mental health at T1. The estimates are adjusted group means on a standardized outcome variable, with a sample mean of 0.

**Table 5:** Change in mental health for high and low-risk subjects

		**High-risk subject**			**Low-risk subject**			
		**N**	**Mean**^**a**^	**CI**	**N**	**Mean**^**a**^	**CI**	**P**
*Women*								.022
	Divorced	118	.51	.36–.67	516	.20	.13–.26	
	Not divorced	1051	.07	.01–.12	11068	-.03	-.05– -.02	
	Total	1169	.12		11584	-.02		
*Men*								.108
	Divorced	122	.67	.49–.86	438	.26	.18–.34	
	Not divorced	1264	.18	.12–.23	8934	-.06	-.07– -.04	
	Total	1386	.22		9372	-.04		

^a^ Mental health at T2 is standardized.

Interaction effects, shown in adjusted group means, between divorce and own alcohol use on change in mental health. Effects are adjusted for age, education, spouses’ alcohol use, and mental health at T1. The estimates are adjusted group means on a standardized outcome variable, with a sample mean of 0.

#### Main effects of time since divorce on change in mental health between T1 and T2

Multivariate analyses of variance, stratified by gender, were conducted with samples restricted to divorced couples. Mental health at T2, adjusted for baseline level at T1, was the outcome variable and time since divorce, categorized as 1–3 years, 4–7 years and 8–10 years since divorce, was the principal predictor. The results, shown in Table 6 were also adjusted for spousal and own alcohol use, age and education. The main effects of time since divorce were significant for both women and men. Women having been divorced 1–3 years had a significantly greater decline in mental health between T1 and T2 than the group having been divorced for the longest period, 8–10 years. The groups having been divorced 4–7 years did not differ significantly from the reference groups.

**Table 6:** Change in mental health according to time since divorce

	**Women**				**Men**			
	**N**	**B**^**b**^	**CI**	**p.**	**N**	**B**^**b**^	**CI**	**p.**
*Time since divorce*				.012				<.000
1–3 yrs	155	.33	.11–.55	.004	145	.68	.40–.96	<.000
4–7 yrs	252	.18	-.01–.38	.069	232	.13	-.12–.38	.313
8–10 yrs	227	0			183	0		

^a^ Mental health at T2 is standardized.

^b^ B = group difference in fractions of standard deviations.

Analysis of variance of time since divorce, adjusted for partners’ and subjects’ alcohol use, age, education and mental health at T1 ^*a*^*.*

#### Interaction effects between time since divorce and spousal and own alcohol use

Interaction terms between time since divorce and spousal alcohol use or subjects’ own alcohol use were specified. The estimates were adjusted for own or spousal alcohol use, the subjects’ age and education and mental health at T1. The results are displayed as simple effects analyses in stratified samples in Table 7 and 8. One interaction effect proved significant – between time since divorce and wives’ high-risk use on husbands’ change in mental health (p = .018). The results suggest that men divorcing high-risk spouses still experience a decline in mental health even 8–10 years after the divorce, whereas the effect of divorce has faded away so many years after divorce for other people (Table 7). In addition, one effect approached significance, between time since divorce and women’s own high-risk use on own change in mental health (p = .062, Table 8).

**Table 7:** Change in mental health according to years since divorce and high or low-risk spouse

		**Divorced from high-risk users**			**Divorced from low-risk users**			
		**N**	**Mean**^**a**^	**CI**	**N**	**Mean**^**a**^	**CI**	**p**
*Women*								.385
	1–3 yrs	31	.69	.31–1.06	124	.41	.22–.60	
	4–7 yrs	42	.22	-.11–.54	210	.32	.18–.47	
	8–10 yrs	62	.11	-.15–.38	165	.14	-.02–.31	
	Total	135	.28		499	.29		
*Men*								.018
	1–3 yrs	35	1.07	.61–1.52	110	.83	.60–1.07	
	4–7 yrs	47	.20	-.20–.60	185	.40	.21–.58	
	8–10 yrs	39	.75	.31–1.19	144	.08	-.13–.28	
	Total	121	.63		439	.40		

^a^ Adjusted group means in standard deviations.

Interaction effects, shown in adjusted group means, between time since divorce and spouses’ alcohol use on change in subjects’ mental health. Effects are adjusted for age, education, subjects’ alcohol use, and mental health at T1. The estimates are adjusted group means on a standardized outcome variable, with a sample mean of 0.

**Table 8:** Change in mental health according to years since divorce and high or low-risk subject

		**Divorced high-risk users**			**Divorced low-risk users**			
		**N**	**Mean**^**a**^	**CI**	**N**	**Mean**^**a**^	**CI**	**p**
*Women*								.062
	1–3 yrs	29	1.03	.55–1.51	126	.33	.16–.55	
	4–7 yrs	53	.65	.28–1.01	199	.24	.09–.44	
	8–10 yrs	36	.15	-.30–.59	191	.12	-.08–.20	
	Total	118	.59		516	.21		
*Men*								.417
	1–3 yrs	37	1.23	.75–1.70	108	.79	.56–1.03	
	4–7 yrs	36	.43	-.06–.91	196	.32	.14–.49	
	8–10 yrs	49	.66	.25–1.08	134	.09	-.12–.30	
	Total	122	.76		438	.36		

^a^ Adjusted group means in standard deviations.

Interaction effects, shown in adjusted group means, between time since divorce and subjects’ own alcohol use on change in mental health. Effects are adjusted for age, education, spouses’ alcohol use and mental health at T1. The estimates are adjusted group means on a standardized outcome variable, with a sample mean of 0.

## Discussion

In accordance with previous research, we found that marital dissolution was related to an increase in mental health problems for all divorced groups. The worsening in mental health for the divorced compared to people remaining married occurred even though people who were going to divorce reported relatively poor mental health also before the divorce. Our results may be taken as support both for the health selection (poor baseline mental health) and social causation models (the divorce causes a further worsening in mental health) [6-8]. To our knowledge, the present study was the first to investigate the effects of divorce on change in mental health in couples where at least one of the spouses had a risky consumption of alcohol prior to the divorce. We hypothesized that the mental health of high risk users would worsen after the divorce, whereas the mental health of the individuals divorcing high risk users could either improve or worsen after the divorce. The results showed that female high-risk users and men divorcing female high-risk users had a significantly higher increase in mental health problems post divorce, compared to female divorced low risk users and their spouses. A similar trend was seen for men with a risky consumption of alcohol. The worsening of mental health after a divorce seemed to last longer in male ex-spouses of female high-risk users than in other divorced men, and the same but not significant trend was observed for divorced men who themselves had a high pre-divorce alcohol consumption. Although the effects were only significant in couples where the woman drank heavily, low statistical power makes us unable to conclude in regards to a true gender difference. The observed similar tendencies for men who drink heavily in fact suggest that there may be similar increased, and perhaps long-lasting effects in both genders.

The hypothesis that divorcing a high-risk user would lead to a decrease in mental health problems was not supported – divorcing a high-risk user appears to be related to increasing mental health problems. Men divorcing high-risk users even showed a stronger increase in problems than did other divorced men. This suggests that the adversities related to divorcing a high-risk user, in addition to adversities related to divorce in general, outweigh the benefits of getting rid of problems associated with spousal high-risk drinking. There could be several reasons for this. The strain related to living with a high-risk using spouse may continue to affect life after the divorce, and in many cases, the contact between the spouses does not end once the divorce is final. For instance, custodial disagreements or limited control over the relationship between ex-spouse and children may affect the other spouses’ mental health more in cases when the ex-spouse drinks heavily. Sometimes alcohol abusing persons left alone may act rather vindictive toward their ex-spouse, perhaps especially when drunk. Also, leaving a person with alcohol problems may undoubtedly sometimes impose feelings of guilt.

Despite common beliefs and previous research, high-risk alcohol use may not in all circumstances be negative. A recent study showed that a high consumption was related to *less* spousal mental distress, once the variation caused by problems directely associated with alcohol abuse (like being critisiced for drinking too much) was accounted for [19]. Thus, a high consumption of alcohol per se may not necessarily be related to mental health problems for the spouse during the marriage, which in turn may make divorcing a high-risk user just as hard, or even harder than divorcing a normal consumer. Our somewhat broad definition of high-risk alcohol use in the present study (top 13.1% of men and 9.3% of women) may entail that not all in fact have developed a problematic relationship to alcohol, but rather are at risk of it. This may to a certain degree have affected our results, in line with the findings in the aforementioned study.

Female high-risk users experienced more of an increase in mental health problems after the divorce compared to the female normal consumers. The same, but non-significant trend was observed in high-risk drinking men. Previous research has found a higher risk of increasing alcohol consumption and a higher risk of developing or relapsing into alcohol abuse for divorced couples [22,23]. Mental distress after a divorce has been found to return to baseline levels only after remarriage [37], and people who drink excessively may have a lower chance of remarrying than people who drink less.

The analyses of time since divorce showed that in general, most of the detrimental effect of divorce takes place during the first years after divorce (1–3 years) and that the effect after 4–7 years is not much worse than after 8–10 years. Apparently most of the healing occurs within the first few years. This finding is in line with results of several previous studies – that have shown increased mental health problems the first few years after the divorce that recedes as time goes by, but stays at an elevated level compared to baseline levels [38-40]. There is, however, a great deal of variability in recovery from mental health problems after the divorce. In one study, about half of the respondents improved with time, whereas about one fourth of the sample got worse [41].

The significant interaction effect between time since divorce and wives’ high-risk use on the husbands’ change in mental health, and the same, but non-significant trend seen for male high-risk users, indicate that the healing process may be particularly slow for these men. During the first years after the divorce, the mental health problems increased dramatically both in the high-risk groups and the reference groups. The mental health problems declined with time, but were significantly higher for men divorcing high-risk users than for the reference groups even 8–10 years after the divorce. The observed gender differences may reflect true variation or random fluctuations, where a true tendency of delayed healing only could be observed in men.

The previous studies that have reported subsiding mental health problems a few years after the divorce, indicate that a divorce may be seen as a temporary crisis in which most people manage to get through on the positive side as time goes by [38,39]. This finding corresponds well with our results for the reference groups, as opposed to those for men from couples with high pre-divorce alcohol consumption. These seemed to experience more of a chronic strain. The same may be true for women. Interestingly, Hetherington [42] found that although most people tend to adjust well to a divorce in time, about 10% report elevated scores on depression more than 10 years post-divorce. Typically, members of this group were troubled by alcohol or drug abuse, other health problems, low self-esteem, low social support, poverty and anti-social behavior. This may indicate that in some cases negative effects of divorce may be cumulative – problems cause more problems to arise. The trends for men from pre-divorce high-risk couples essentially showed the same pattern. These men (and possibly women) may be particularly vulnerable for the adverse effects of divorce on mental health, causing the healing process to take longer time.

### Methodological considerations

The present study is to our knowledge the first to investigate how spouses of high-risk users or the high-risk users themselves reacts to divorce in terms of change in mental health problems. We were able to investigate this due to our large and representative sample of approximately 12,000 couples with two-wave questionnaire data with an 11-year interval, and registry based data on marital status and time of change in marital status.

However, there are methodological limitations to our study. Only 38.0% of the invited couples had returned all four questionnaires in both HUNT1 and 2. This may have caused a selection bias. However, an attrition study of the HUNT 2 sample showed that high alcohol consumption and mental distress in HUNT 1 only predicted non-participation in HUNT 2 moderately well (alcohol consumption: OR = 1.27 for the top 3% consumption; mental distress: OR = 1.84 for the top 1%) [43]. Divorce was a stronger prediction of non-participation in HUNT 2 (OR = 1.98). In general, even highly selective non-participation are not usually expected to influence associations between variables dramatically [44], giving reason to believe that our estimates have not been severely affected by non-participation. However, if people with the strongest loss of mental health from T1 to T2 tend to drop out of the study, and if this selection effect is stronger among divorced people than among people who are still married, the observed association between divorce and mental health problems may be somewhat attenuated. Correspondingly, if such a selection effect is stronger among alcohol burdened divorcing couples than among other divorcing couples, the observed difference between divorcees from alcohol burdened couples and other divorced people will be attenuated. However, we believe that the risk of such a differential selection effect is small.

Due to few divorced couples where both spouses had participated at T1 and T2, we were compelled to shift the focus from alcohol abuse to heavy risk use in order to maximize the number of divorced cases with a (likely) alcohol problem. The top 13% of the men and top 9% of the women were categorized as high-risk users in our study. The male heavy risk use falls within the 12-month prevalence rates for alcohol use disorders among men (16%), whereas the female group is somewhat larger than the 12-month prevalence rates for women (6%) [45]. Compared to the affirmative response percentages on the alcohol frequency, influence of alcohol and excessive drinking items of the low-risk groups, the high-risk men and women clearly exhibit drinking behaviors representing a risky consumption. However, the broadly defined high-risk categories may have somewhat attenuated our effect estimates, especially for women.

The 11 year time gap between T1 and T2 makes us able to investigate long term effects of divorce. However, a lot of events may have taken place during this long period. People may remarry, start new families, even both remarry and divorce, get somatically ill or experience other kinds of events that may impact the mental health in a positive or negative way at T2. Remarriage is related to improvements in mental health [37], and as some of the respondents divorced between T1 and T2 are bound to have remarried before T2, the inclusion of these may have underestimated the effects of divorce on change in mental health.

The mental health index used in the present study was based on nine items asking about a variety of symptoms indicative of general mental health problems. As the measure has not been broadly validated, there is a risk of unsatisfactory construct validity of the outcome measure. However, the high correlation with the Hopkins Symptom Checklist [35] - a well validated measure of mental distress – speaks in support of the validity of the measure. Rather than using the term “mental distress”, we chose the more general term “mental health problems” due to the wide variety of symptoms tapped by the nine items.

Perhaps the largest methodological weakness of our study is the small groups in the interaction analyses of time since divorce and heavy risk alcohol use. The small number of observations and the wide confidence intervals imply that the results from the analyses of time since divorce should be interpreted with caution. Especially a significant observed effect in one gender only cannot be taken as evidence that the same effect does not exist in the other gender as well. The results need to be replicated in a larger sample.

Besides of random errors, our results could be systematically biased due to confounding between alcohol consumption and mental health problems from sources not adjusted for. For instance heavy drinkers might tend to marry persons with mental health problems. We would expect such confounding to primarily affect the baseline mental health problems, not the estimates of change in mental health. But we also cannot rule out the possibility of confounding from sources of vulnerability that make people from alcohol risk couples respond more strongly to divorce than do other people. Such confounding would inflate the observed differences between divorced subjects from alcohol exposed couples and other divorced subjects.

## Conclusions

The present study is the first to investigate the effects of divorce on change in mental health among couples where at least one of the spouses have a risky consumption of alcohol before the divorce. Individuals who will get divorced have more mental health problems prior to the divorce. In particular high-risk users suffer prior to the divorce. However, the divorce leads to additional strain for both normal consumers and high-risk users - and their spouses. Female high-risk users and their husbands experienced a significantly higher increase in mental health problems post divorce compared to female divorced low-risk users and their husbands. Similar trends were seen for high-risk using men. The time of the healing process seems to vary for men from couples with high pre-divorce alcohol consumption, as the increase in mental health problems lasted for as long as 8–10 years after the divorce. Although the same trend was not observed for women from couples with high pre-divorce alcohol consumption, random fluctuations may have obscured a similar relationship among women. Our results need to be replicated. If they are, health care professionals should be aware of the difference in increase and healing process, and couples with a high pre-divorce alcohol consumption may benefit from additional health care after the divorce in order to lower the chance of chronic strain.

## Competing interest

The authors declare that they have no competing interests.

## Authors’ contributions

KR was responsible for the design, carried out the statistical analyses and drafted the manuscript. FAT and MI contributed to the design and revision of the manuscript. KT contributed by designing the study, methodological supervision and revising and drafting the manuscript. All authors read and approved the final manuscript.

## Pre-publication history

The pre-publication history for this paper can be accessed here:

http://www.biomedcentral.com/1471-2458/13/852/prepub
